# Factors influencing the ownership and utilization of long-lasting insecticidal nets for malaria prevention in Ethiopia

**DOI:** 10.1186/s12936-017-1907-8

**Published:** 2017-07-01

**Authors:** Admasu Tassew, Richard Hopkins, Wakgari Deressa

**Affiliations:** 1 Ethiopian Institute of Water Resources, Addis Ababa University, Akaki Campus, P. O. Box 150461, Addis Ababa, Ethiopia; 2 Department of Biology, Arba Minch University, Arba Minch, Ethiopia; 3 Natural Resources Institute, University of Greenwich, Chatham Maritime, UK; 4 Department of Preventive Medicine, School of Public Health, College of Health Sciences, Addis Ababa University, Addis Ababa, Ethiopia

**Keywords:** LLIN, Multivariable logistic regression, Ownership, SSA, Utilization

## Abstract

**Background:**

Utilization of long-lasting insecticidal nets (LLINs) is regarded as key malaria prevention and control strategy. However, studies have reported a large gap in terms of both ownership and utilization particularly in the sub-Saharan Africa (SSA). With continual efforts to improve the use of LLIN and to progress malaria elimination, examining the factors influencing the ownership and usage of LLIN is of high importance. Therefore, the current study was conducted to examine the level of ownership and use of LLIN along with identification of associated factors at household level.

**Methods:**

A cross-sectional study was conducted in Mirab Abaya District, Southern Ethiopia in June and July 2014. A total of 540 households, with an estimated 2690 members, were selected in four kebeles of the district known to have high incidence of malaria. Trained data collectors interviewed household heads to collect information on the knowledge, ownership and utilization of LLINs, which was complemented by direct observation on the conditions and use of the nets through house-to-house visit. Bivariate and multivariable logistic regression analyses were used to determine factors associated to LLIN use.

**Results:**

Of 540 households intended to be included in the survey, 507 responded to the study (94.24% response rate), covering the homes of 2759 people. More than 58% of the households had family size >5 (the regional average), and 60.2% of them had at least one child below the age of 5 years. The ownership of at least one LLIN among households surveyed was 89.9%, and using at least one LLIN during the night prior to the survey among net owners was 85.1% (n = 456). Only 36.7% (186) mentioned at least as the mean of correct scores of all participants for 14 possible malaria symptoms and 32.7% (166) knew at least as the mean of correct scores of all participants for possible preventive methods. Over 30% of nets owned by the households were out of use. After controlling for confounding factors, having two or more sleeping places (adjusted odds ratio [aOR] = 2.58, 95% CI 1.17, 5.73), knowledge that LLIN prevents malaria (aOR = 2.51, 95% CI 1.17, 5.37), the presence of hanging bed nets (aOR = 19.24, 95% CI 9.24, 40.07) and walls of the house plastered or painted >12 months ago (aOR = 0.09, 95% CI 0.01, 0.71) were important predictors of LLIN utilization.

**Conclusions:**

This study found a higher proportion of LLIN ownership and utilization by households than had previously been found in similar studies in Ethiopia, and in many studies in SSA. However, poor knowledge of the transmission mechanisms and the symptoms of malaria, and vector control measures to prevent malaria were evident. Moderate proportions of nets were found to be out of use or in poor repair. Efforts should be in place to maintain the current rate of utilization of LLIN in the district and improve on the identified gaps in order to support the elimination of malaria.

## Background

Long-lasting insecticidal nets (LLIN) are regarded as a key weapon in the armory of effective malaria vector preventive measures, and hence malaria prevention strategies [1]. The World Health Organization (WHO) has reported this as a success story contributing to the decline in the number of malaria cases at the end of 15 year Millennium Development Goals (MDGs) period. Accordingly, out of the total 663 million malaria cases averted in the past 15 years in sub-Saharan Africa (SSA), 67–73% has been attributed to the extensive distribution and use of LLIN and insecticide-treated nets (ITNs) [1].

Malaria is one of major public health problems in Ethiopia; about 68% of Ethiopians are at risk of the disease [2], 27% at high risk (>1 case per 1000 population), 41% at low risk (0–1 case per 1000 population) [1]. Parasite positivity rate in malaria endemic areas (<2000 m) is 1.3% for microscopy, whereas rapid diagnostic test (RDT) results showed 4.5% parasite positivity rate [3].

Long-lasting insecticidal nets (LLIN) are important preventive measure deployed in Ethiopia along with indoor residual spraying (IRS), environmental management and larval control [4]. With free mosquito net distribution in some countries including Ethiopia, universal coverage might be assumed to be attainable. However, there is large gap both in terms of ownership and utilization particularly in SSA where 90% of the malaria burden exists. Ownership of LLIN/ITN ranges between 34 and 98.4% of households at risk of malaria in SSA [5–13]. The utilization trend in the same region has even higher variation although net use is becoming more commonplace with time. Among households which claim to own at least one LLIN/ITN, the utilization rate was found to be from 33.5 to 69% in SSA [10, 13–16]. In addition, a considerable proportion of the nets in use have been reported to be a poor condition [9].

Several factors have been suggested to influence the ownership and use of LLIN/ITN, given that the intervention is effective. Ownership of bed nets has been found to be positively associated to the socio-economic status of a household, the age of the household head, (women’s) knowledge of malaria, and the presence of a pregnant woman or children below the age of 5 years [7–9, 17]. Increasing distance to the nearest health facility and poor access to transport service, on the other hand, have been reported to negatively affect net ownership [9, 17]. The distribution strategy for bed nets can also be a major factor, with targeted distribution of LLIN in Uganda increasing the ownership of at least one net by 47% [11]. Prior positive experience of net use and awareness on the benefits, level of education, family size, and monthly income were among factors associated with net use in the SSA [5, 18]. Studies from Ethiopia in particular indicated sex, age difference, distance of a house from breeding site, number of separate sleeping rooms, preferred net colour by a household and number of nets owned to be good predictors of LLIN use [10, 13]. Targeted behavioural change communication was also testified to considerably improve utilization [19, 20].

Given LLIN/ITN is the major intervention to reduce the burden of malaria, it is imperative to assess the level of LLIN ownership and utilization among the people at risk. The current study determines some of the factors which influence LLIN ownership, and utilization at the household level in a highly malarious region of Ethiopia.

## Methods

### Study setting

This study was conducted in Mirab Abaya District, Gamo Gofa Zone—Southern Ethiopia, 465 km south west of Addis Ababa. The district is located between 6.11°–6.51 °N and 37.58°–37.98 °E bordering Lake Abaya to the east, Chencha, and Boreda districts to the west, Boreda district and Wolaita zone to the north, and Arba Minch Zuria district to the south. The district has 24 *kebeles* (kebele is the smallest administrative unit in Ethiopia) of which 23 are rural kebeles and the remaining one is urban. Malaria is endemic to the urban and 16 of the rural kebeles. The district had an estimated population 92,540 (46,223 males and 46,317 females) in 2014. About 67.5% (62, 493) of the district’s population live in 16 malarious lowland kebeles. There are four health centers in the district of which two are in the malarious kebeles. A further one or two health posts in each kebele constitute the primary health care system along with the health centers linked through a referral system. Distribution of LLIN, indoor residual spraying (IRS) and environmental management campaigns are the major malaria vector prevention/control interventions in the area.

The major socio-economic activity in the study area is mixed farming, of which crop production is the leading component. The presence of small scale irrigation schemes to sustain crop production throughout the year make the study area important focus for malaria research along with its proximity to the shore of Lake Abaya.

### Sample size determination

In Ethiopia, LLIN are distributed free, and consequently the ownership and use of such nets was assumed to be universal. This study is part of a wider spatio-temporal investigation of malaria and associated factors in the area. Accordingly, a community based cross-sectional survey was conducted in June 2014. The sample size for this study was determined based on a single population proportion formula. Taking a recent finding of about 69% utilization of LLIN by households in a comparable area in Ethiopia [13], 95% confidence level and 5% precision, the required sample size was 327 households. The final minimum sample size required for the study was 540 households after considering a design effect of 1.5 and 10% non-response rate.

### Sampling procedure

The households in the malarious kebeles constituted the source population. The sampling technique used was multistage sampling with kebeles and households as primary and secondary sampling unit respectively. Four of the 16 rural malarious kebeles in the district were randomly drawn from which the households were systematically selected. The allocation of the sample households to the kebeles was proportional to the kebele size. The lists of households in each of the study kebeles were obtained before undertaking systematic sampling to draw the study households. The sampling interval was established based on the total number of households in the kebeles. Households were substituted with the nearest neighbour in cases of refusal to participate or if the house was not inhabited. The selected households in each kebele were given unique identification numbers.

### Data collection methods

The data collection was purposely set in the peak malaria transmission period following the rainy season (March–May). A total of seven data collectors with a college level diploma graduates and resident in the respective kebeles were recruited. All the data collectors were fluent speakers of the local languages. Efforts were made to maintain data quality pre-, during and post data collection. Prior affiliation of the data collectors to health care givers was checked during recruitment to minimize information bias. Training was offered on the basics of research with an emphasis on ethical issues, the data collection tool prepared (questionnaire) and how to administer it followed by a pre-test. The pre-test was conducted on households in kebeles of similar setting with the study area. The data collection process was closely supervised and the completeness of the required information was confirmed.

The questionnaire consisted of major parts such as socio-demographic information, knowledge on the magnitude, transmission, sign and symptom of malaria, knowledge and practice of malaria prevention, ownership and utilization of mosquito nets and a description of the house. Household heads were interviewed with the prepared questionnaire. Household conditions and complementary information on the interview were observed and recorded (e.g., eaves, condition of the building, inspecting hanged nets, condition of the net).

### Study variables

The outcome variable for this study is LLIN utilization defined as the use of at least one LLIN by the households during the night prior to the survey. Important predictors of the outcome variable include socio-demographic factors, knowledge and perceptions related to malaria, ownership, preference and condition of LLIN along with others. A knowledge level of symptoms of malaria was calculated based on the number of correct answers for symptoms of malaria: fever, shivering, sweating, headache, vomiting, loss of appetite, bitterness of mouth, weakness, splenomegaly, back pain, anaemia, convulsion, thirsty and joint pain plus any others mentioned by the respondents. A score of one was given to “yes” response for each of the options. The average score of all the respondents (sum of scores divided by the number of respondents) was taken as a cut-point to categorize an individual respondent to “knowledgeable” or otherwise. Similarly, knowledge of preventive methods against malaria was defined based on: using LLIN, spraying insecticide, draining mosquito breeding sites, spraying aerosol plus any other added by the respondents. Painting or plastering of the wall of a house within or before 12 months has an implication on the potency of IRS, thereby influencing LLIN use through its effect on indoor mosquito abundance.

### Data analysis

The data collected was checked for completeness and entered into Epi-Info version 3.5.3 (Center for Disease Control and Prevention of the United States of America (CDC), 2011, Atlanta-Georgia). It was then exported to SPSS version 20 (IBM Corp., 2011, Armonk-New York) for data cleaning and analysis. Frequencies and proportions were calculated for socio-demographic characteristics, and knowledge, perception and practices related to malaria transmission and prevention. Separate descriptive statistics on preference, ownership and utilization of LLINs were performed. Ownership of LLIN was calculated as a proportion of households having at least one LLIN out of the total households surveyed. Once the ownership of LLIN was identified; the data of LLIN owner households was separately treated to determine the utilization and associated factors. Utilization of LLIN was estimated based on whether households have used at least one LLIN the night before the survey took place.

Bivariate logistic regression analysis was performed to identify factors associated with the outcome variable of interest, LLIN utilization (use versus non-use during the night prior to the survey). Factors with *p* value *≤*0.2 were selected as candidates for the final model. The independent contribution of the selected variables on the outcome (LLIN utilization) was analyzed using a multivariable logistic regression model with the level of significance is at p < 0.05. Crude odds ratio (cOR) and adjusted odds ratio (aOR) were presented.

### Ethical considerations

The ethical clearance for the study was obtained from the ethical review committee of College of Medicine and Health Sciences, Arba Minch University. The data was collected based on the informed consent of the participants. All participants were clearly informed about the nature, purpose, and benefits of the survey. The right to decline to participate as well as to decline to answer individual questions was made clear. Formal letters with the study objectives were sent to authorities at Gamo Gofa Zone Health Department, Mirab Abaya District Health Office and the administration offices of the kebeles involved.

## Results

### Socio-demographic characteristics of the respondents

A total of 507 households responded to the study (94% response rate), where a total of 2759 people were living. More than 58% (296) of the households had a family size >5, which is above the average for southern Ethiopia and 60% (305) of the households had at least one child below 5 years of age. Few pregnant women were encountered in this study, only in 28 (5.5%) of the households surveyed. Approximately 33.7% of the participants can’t read or write and just over three-quarters were farmers. The proportion of selected socio-demographic characteristics of the participant households is shown in Table 1.

**Table 1:** Socio-demographic characteristics of respondents, Mirab Abaya District, Southern Ethiopia, 2014

Characteristics	Sex		
	Male, n (%)	Female, n (%)	Total, n (%)
Age in years			
17–24	13 (4.3)	20 (9.9)	33 (6.5)
25–40	156 (51.1)	109 (54.0)	(52.3)
41–60	80 (26.2)	57 (28.2)	(27.0)
>60	56 (18.4)	16 (7.9)	72 (14.2)
Religion			
Protestant christian	265 (86.9)	170 (84.2)	(85.8)
Orthodox christian	36 (11.8)	30 (14.9)	(13.0)
Others	4 (1.3)	2 (1.0)	6 (1.2)
Ethnicity			
Gamo	158 (51.8)	88 (43.6)	(48.5)
Wolaita	108 (35.4)	82 (40.6)	(37.5)
Gidicho	38 (12.5)	30 (14.9)	67 (13.4)
Others	1 (0.3)	2 (1)	3 (0.6)
Maital status			
Married	299 (98.0)	132 (65.3)	431 (85.0)
Widower/ed	4 (1.3)	62 (30.7)	66 (13.0)
Never married/divorced/separated	2 (0.7)	8 (4.0)	10 (2.0)
Level of education			
Can’t read or write	55 (18.0)	68 (33.7)	123 (24.3)
Can read and write only	59 (18.3)	28 (13.9)	87 (17.2)
Primary education (1st cycle)	56 (18.4)	29 (14.4)	85 (16.8)
Primary education (2nd cycle)	84 (27.5)	35 (17.3)	119 (23.5)
Secondary education	39 (12.8)	17 (8.4)	56 (11.0)
Others	12 (3.9)	25 (12.4)	37 (7.3)
Main occupation			
Farmer	242 (79.3)	136 (67.3)	378 (74.6)
Housewife		36 (17.8)	36 (7.1)
Labourer	26 (8.5)	8 (4.0)	34 (6.7)
Others	37 (12.2)	22 (10.9)	59 (11.6)

### Knowledge, perception and practice related to malaria

Nearly 90% (441) of the respondents perceived malaria as the top health problem in the study area. Out of the total 507 households in this survey, 441 (87.3%) associated mosquito bites with malaria infection. There was a very high understanding of the importance of bed nets, 99.2 and 97.6% of the respondents knew the importance of LLIN for prevention of mosquito bites and malaria respectively. The wider benefits of bed nets use were also understood; with 86.2% (437) of respondents mentioning that LLIN prevents the bites of other insects. However, responses on knowledge of the symptoms and prevention methods of malaria were widely varied. Over 80% (406) of respondents identified fever as symptom of malaria, whereas less than 2/3rd (316) associated malaria with shivering and chill (Table 2).

**Table 2:** Knowledge, perception and practice related to malaria magnitude, transmission and prevention in Mirab Abaya District, 2014

Characteristics	Frequency, n (%)
Malaria is top health problem (n = 507)	
Yes	455 (89.7)
No	52 (10.3)
Infection is by mosquito bite (n = 505)	
Yes	441 (87.3)
No	64 (12.7)
Mosquito bite high	
Evening	435 (85.8)
Night	122 (24.1)
Day	23 (4.5)
Using LLIN prevents mosquito bite	
Yes	503 (99.2)
No	4 (0.8)
Using LLIN prevents malaria (n = 499)	
Yes	487 (97.6)
No	6 (1.2)
Don’t know	6 (1.2)
Malaria is curable (n = 503)	
Yes	500 (99.4)
No	1 (0.2)
Don’t know	2 (0.4)
First line treatment drug (n = 504)	
ACT	433 (85.9)
Chloroquine	29 (5.7)
Other	25 (5.0)
Don’t know	17 (3.4)
Knowledge on symptoms	
Fever (n = 506)	406 (80.2)
Shivering/chill (n = 505)	316 (62.6)
Headache (n = 506)	286 (56.5)
Loss of appetite (n = 506)	161 (31.8)
Vomiting (n = 507)	147 (29.0)
Back pain (n = 506)	100 (19.8)
At least one symptom (n = 507)	503 (99.3)
Aggregate knowledge^a^ (n = 507)	186 (36.7)
Knowledge on prevention	
Using mosquito net (n = 506)	415 (82.0)
Clean house (n = 485)	331 (68.2)
Eating good food (n = 485)	302 (62.3)
Drain breeding sites (n = 485)	174 (35.9)
Spraying insecticides (n = 485)	57 (11.8)
Others (n = 490)	47 (9.6)
At least one method (n = 507)	463 (91.3)
Aggregate knowledge*(507)	166 (32.7)
Preventive practices	
Use mosquito net (n = 490)	429 (87.6)
Drain breeding sites (n = 492)	104 (21.1)
Use aerosol (n = 489)	77 (15.7)
Close doors/windows (n = 491)	66 (13.4)
Others (n = 491)	64 (13.0)

^a^Refers to the proportion of respondents answered at least as the mean score of total respondents for the options of the given question which was 4 out of 9 for knowledge of symptoms, and 2 out of 4 for knowledge of preventive methods

The proportion of respondents noted other symptoms of malaria were 11% (sweating), 10.7% (bitterness of mouth), 7.9% (thirst), 6.9% (splenomegaly), 6.1% (convulsion), 5.5% (weakness) and 2% (joint pain and anaemia). For all possible symptoms of malaria aggregated, only 36.7% (186) of the respondents answered at least as the mean of correct score of all participants.

Bed net use was frequently mentioned as a major preventive method against malaria and also the leading method of prevention in practice too. The preventive practices of the respondents were ranked in descending order from use of mosquito net, through draining mosquito breeding sites, use of insecticide aerosols and closing doors and windows early in the evening. Other practices mentioned include smoking dung or leaves to repel mosquito (32), blocking mosquito entry holes (29), and using garlic (3).

### Factors associated with ownership of LLINs

From the total households surveyed, 89.94% (456) had at least one LLIN during the survey. The total number of nets available (used and unused) within these households was 945, putting an average of 2 nets per household regardless of family size. The proportion of nets actually in use was only moderate, 69.5% (657) of the nets in the households. Nets were generally in good condition, but 10.3% (97) of the nets available were found to have holes that could allow entry of mosquito.

Mosquito net distribution in Ethiopia is free of charge and hence exploring factors for acquisition may not be important; more than 98% (448) of LLIN owner households received the nets from public health facilities in their kebele of residence. The time gap between net distribution and the survey could contribute to the variation in ownership because retention of the received bed nets can be dictated by a number of variables. Bivariate logistic regression was run to explore factors associated with the ownership of at least one LLIN during the survey. Respondents’ level of education, knowledge that a bed net prevents malaria, type of sleeping place and the presence or absence of cover/curtain for windows were significantly associated with LLIN ownership (*p* < 0.05). Sleeping on a bed frame and having a curtain or any kind of cover for the windows was likely to be linked to a failure to own LLIN in the study area. However, opening at the eaves of the houses that can allow entry of mosquito did not indicate significant association with LLIN ownership. Age and sex of respondents were also included in the bivariate analysis.

Predictors of LLIN ownership from the bivariate logistic regression analysis with at least marginal significance of p ≤ 0.2 were fitted to multiple logistic regression model. Five variables showed statistically significant association with the outcome of interest at p < 0.05 (Table 3). The age of respondents between 26 and 40 and 41–60 were 3.3–3.4 times more likely to possess LLINs compared to those respondents whose age was either under 26 or over 60 years (aOR = 3.3, 95% CI 1.24, 8.81; aOR = 3.4, 95% CI 1.13,10.24 respectively). The level of education of respondents also showed significant positive association with ownership of LLINs by the households surveyed. Respondents’ knowledge on LLIN as preventive to malaria was also significantly associated with ownership of LLIN p < 0.05 (aOR = 2.39, 95% CI 1.23, 4.65). Possession of bed frame, however, showed negative significant association compared to other sleeping place options such as ground or flat form (“medeb”) (aOR = 0.50, 95% CI 0.26, 0.90). Use of curtain/cover for the window was also likely to hinder bed net ownership (Table 3).

**Table 3:** Factors independently associated with LLIN ownership among households in Mirab Abaya District, Southern Ethiopia, 2014

Variable	Households used LLIN (n, %)	aOR (95% CI)	p value
Age of respondent (years)			
>60	72 (14.2)	1.52 (0.50, 4.58)	0.461
41–60	137 (27.0)	3.40 (1.13, 10.24)	0.030*
26–40	247 (48.7)	3.30 (1.24, 8.81)	0.017*
≤25	51 (10.1)	1	
Level of education			
Secondary or above	93 (18.3)	2.57 (0.99, 6.67)	0.053
Second cycle primary	119 (23.5)	4.46 (1.56, 12.70)	0.005*
First cycle primary	85 (16.8)	1.34 (0.57, 3.18)	0.503
Can read and write	87 (17.2)	3.40 (1.17, 9.94)	0.025*
Can’t read or write	123 (24.3)	1	
Sleeping under net prevents			
Yes	415 (81.9)	2.39 (1.23, 4.65)	0.010*
No	92 (18.1)	1	
Sleeping place option			
Bed frame	396 (78.1)	0.50 (0.26, 0.90)	0.035*
Other	111 (21.9)	1	
Windows have curtain/cover			
Yes	153 (30.2)	0.48 (0.26, 0.95)	0.023*
No	354 (69.8)	1	

*aOR* adjusted odds ratio

* Significantly associated at p < 0.05

### Factors associated with utilization of LLINs

Amongst 456 households owning LLIN, 85.1% (388) had used at least one LLIN the night before the survey took place. Of the households reported to possess LLIN, 83.6% (381) kept at least one bed net hanging, whereas 16.4% (75) did not hang any bed net. Bivariate logistic regression analysis was performed to select candidate predictors for net utilization. Knowledge of the symptoms of malaria was not significantly associated with LLIN utilization, whereas perceiving malaria as the top health problem, knowledge that mosquito bites can transmit malaria and sleeping under LLIN prevents it, and knowledge of preventive methods showed significant association at p < 0.05. The number of sleeping places in the house, preferred net shape and colour, hanging LLIN and the number of months ago the wall of a house was painted/plastered are also likely predictors of LLIN utilization (p < 0.05). Presence of two or more sleeping places was observed to enhance utilization of nets, thereby allow more under five children to sleep under a LLIN. Households’ use of net was also directly related with the shape and colour of preference, and number of nets hanged. Despite the statistical eligibility, the majority of those variables explained small amount of variations in the outcome (R^2^ = 0.025 to 0.072) except for hanging nets which accounts for up to 49.8% of the variations (R^2^ = 0.498). Socio-demographic variables such as age categories, sex and level of education of respondent as well as family size did not show significant association to LLIN utilization at p < 0.05 (Table 4).

**Table 4:** Factors associated with LLIN utilization the night preceding the survey among households in Mirab Abaya District, Southern Ethiopia, 2014

Variable	Households used LLIN (n, %)	cOR (95% CI)	p value
Age of respondent (years)			
>60	60 (13.16)	0.81 (0.27, 2.43)	0.708
41–60	124 (27.19)	1.18 (0.43, 3.26)	0.752
26–40	229 (50.22)	0.84 (0.33, 2.14)	0.717
≤25	43 (9.43)	1	
Sex of respondent			
Male	271 (59.43)	1.57 (0.94, 2.63)	0.088
Female	185 (40.57)	1	
Level of education			
Secondary or above	84 (18.42)	2.16 (0.93, 5.01)	0.073
Second cycle primary	113 (24.78)	1.83 (0.88, 3.83)	0.107
First cycle primary	75 (16.45)	1.24 (0.57, 2.66)	0.587
Can read and write	82 (17.98)	1.67 (0.76, 3.71)	0.205
Can’t read or write	102 (22.37)	1	
Number of sleeping place			
Three or more	156 (34.21)	2.22 (1.14, 3.31)	0.018*
Two	198 (43.420	2.13 (1.15, 3.96)	0.017*
One	102 (22.37)	1	
Malaria is top health problem			
Yes	408 (89.5)	2.38 (1.19, 4.79)	0.015*
No	48 (10.5)	1	
Mosquito bite infection route			
Yes	398 (87.3)	2.27 (1.18, 4.37)	0.014*
No	58 (12.7)	1	
Family member with fever			
Yes	52 (11.4)	0.96 (0.43, 2.14)	0.919
No	404 (88.6)	1	
Knowledge of symptoms			
Good	181 (39.69)	1.57 (0.90, 2.73)	0.109
Poor	275 (60.31)	1	
Knowledge of preventive methods			
Good	410 (89.91)	2.54 (1.26, 5.13)	0.009*
Poor	46 (10.09)	1	
Sleeping under net prevents			
Yes	382 (83.8)	2.58 (1.42, 4.68)	0.002*
No	74 (16.2)	1	
Months ago net received			
1–12	340 (74.6)	0.53 (0.22, 1.29)	0.160
13–24	57 (12.5)	3.11 (0.60, 16.12)	0.176
>24	59 (12.9)	1	
Preferred net shape			
Rectangular	296 (64.9)	4.21 (2.17, 8.18)	0.000*
Conical	101 (22.1)	2.05 (0.98, 4.30)	0.057
Any shape	59 (13.0)	1	
Preferred net colour			
White	76 (16.7)	1.57 (0.71, 3.48)	0.265
Green	58 (12.7)	1.34 (0.58, 3.08)	0.494
Blue	261 (57.2)	4.00 (1.98, 8.09)	0.000*
Any colour	61 (13.4)	1	
Net hanged			
Two	129 (28.3)	27.55 (10.78, 70.42)	0.000*
One	252 (55.3)	16.45 (8.57, 31.70)	0.000*
None	75 (16.4)	1	
People slept last night at home			
1–5	215 (47.1)	0.66 (0.39, 1.11)	0.119
>5	241 (52.9)	1	
Holes on nets for mosquito entry			
Yes	71 (15.6)	1.35 (0.69, 2.62)	0.383
No	385 (84.4)	1	
Months ago IRS conducted			
>12	123 (27.0)	0.94 (0.53, 1.68)	0.846
≤12	333 (73.0)	1	
Months ago wall painted/plastered			
>12	394 (86.4)	0.08 (0.01, 0.59)	0.013*
≤12	62 (13.6)	1	

*cOR* Crude odds ratio

* Significantly associated at p < 0.05

A multiple logistic regression was performed to identify factors which independently predict of LLIN utilization the night preceding the survey. Accordingly, the number of sleeping places, knowledge that LLIN prevents malaria, number of months ago the wall of the house was plastered or painted and hanging bed nets are likely predictors of LLIN utilization at p < 0.05. The availability of more than two sleeping places for a household has exhibited strong positive association with LLIN use at p < 0.01. The hanging of at least one mosquito net by a household showed the strongest positive statistical association with net utilization at p < 0.001. Households whose wall was painted or plastered more than 12 months prior to the survey were made less use of LLIN compared to those households whose walls were painted or plastered within the last 12 months. The factors independently associated with mosquito net utilization in this study accounted for up to 58% of the variations in LLIN utilization among the households the night prior to the survey in the study area (Table 5).

**Table 5:** Factors independently associated with LLIN utilization the night preceding the survey among households in Mirab Abaya District, Southern Ethiopia, 2014

Variable	Households used LLIN (n, %)	aOR (95% CI)	p value
Number of sleeping place			
Three or more	156 (34.21)	3.98 (1.66, 9.58)	0.002
Two	198 (43.420	2.58 (1.17, 5.73)	0.020
One	102 (22.37)	1	
Sleeping under net prevents			
Yes	382 (83.8)	2.51 (1.17, 5.37)	0.018
No	74 (16.2)	1	
Net hanged			
Two	129 (28.3)	23.62 (8.98, 62.13)	0.000
One	252 (55.3)	19.24 (9.24, 40.07)	0.000
None	75 (16.4)	1	
Months ago wall painted/plastered			
>12	394 (86.4)	0.09 (0.01, 0.71)	0.023
≤12	62 (13.6)	1	

*aOR* adjusted odds ratio

## Discussion

The findings of the present study provide an important account on the utilization of LLIN and the factors which are associated with their use at household level. Out of total 456 households owning at least one mosquito net, 388 (85.1%) used it in the night prior to the survey. This proportion was higher compared to the findings of similar studies in Ethiopia [10, 13, 21] and across SSA [12, 22–24]. This could be because of the additional affirmative information as part of the effort to attain the 2015 target as per the national malaria guideline [4] since the earlier studies were conducted. According to the guideline training, education and sensitization at community level was put forward as an important strategy to attain higher LLIN ownership and utilization. The presence of nets hanged, the number of sleeping places, and knowledge that sleeping under bed nets prevents malaria transmission were important predictors positively associated with LLIN utilization. The presence of hanging nets was the strongest predictor of high LLIN utilization among net owning households in this study. This is in harmony with previous studies conducted in Ethiopia [25, 26]. Similarly, a study from Botswana indicated that presence of hanging nets was a major predictor of LLIN use by households [24]. The proportion of net owner households hanging at least one LLIN in the present study was 83.6%, which was higher compared to earlier findings across SSA [27, 28].

The number of sleeping places, another strong predictor of LLIN use, was positively related to larger families in the current study. The association of more sleeping places to larger families was also reported by Woyessa et al. [25]. Larger families among net owners in this study showed to have more children under 5 years of age, 169 households with family size >5 versus 110 households with family size ≤5. Consequently, the number of sleeping places was associated with more people, thereby more children sleeping under net.

The respondents’ knowledge that sleeping under a net prevents the transmission of malaria predicted both the ownership and utilization of LLIN by households. About 84% of LLIN user households associated sleeping under net with malaria prevention in the current study. This is higher compared with previous studies in Ethiopia [16, 29]. The participants of the current study have also demonstrated a better knowledge of malaria preventive mechanisms in general compared to participants involved in studies conducted in other parts of SSA [6, 30].

Higher LLIN utilization was also evident among households whose wall was painted or plastered within the 12 months preceding the survey. This could be because painting or plastering the wall of houses could reduce or remove the effect of indoor residuals sprayed, thereby allow more indoor mosquitoes. Indoor residuals spray (IRS) in malaria endemic settings such as the study area of the current research are meant to control seasonal transmission of the disease [4]. The main indoor residual spraying schedule in the study area was around August; roughly 12 months prior to the survey.

The proportion of LLIN owner households (89.94%) in this study was found to be higher compared to similar studies in Ethiopia [10, 13, 16] and Africa [5, 11, 30]. The higher proportion of LLIN ownership could be because of additional distribution of nets in recent years. Important factors significantly associated with ownership of LLIN included age category of respondents, level of education, knowledge that sleeping under a mosquito net prevents malaria, type of sleeping place and presence/absence of window curtain/cover were. Comparatively significant association of responses by age groups from 26–40 to 41–60 could be because the proportions of respondents in these groups are considerably higher in numerical terms (nearly 76% of the respondents).

The significant association between level of education and net ownership by households was also reported in another study [25]. Higher level of education affirmatively influences the knowledge of linking sleeping under net with malaria prevention [6]. In this study, the knowledge of respondents that sleeping under net prevents malaria transmission positively predicted net ownership. This is an important factor, which can be upgraded through behavioural change communication to improve LLIN ownership further [20]. Sleeping on a bed frame and having curtain/cover over windows showed significant negative association with mosquito net ownership. The negative association with sleeping on a bed frame may be due to the fact that considerable proportion (86.2%) of respondents considered LLIN as mechanical protection thereby enhanced its usage on other sleeping place options such as ground or flat form (“medeb”). Obviously, curtain or cover for windows if appropriately used on the right time could minimize number mosquitoes indoor. This could be the reason for the low ownership of LLIN among households having window curtain/cover.

Generally, this survey was conducted following the main rainy season in the study area when mosquitoes were abundant. Consequently, such a reason should be taken into account in interpreting the findings as the season might have encouraged the people to use the nets more. Outcomes in ownership as well as LLIN utilization could show different proportions if studies conducted in other seasons of the year. As strength of this study, inspection by direct observation has been conducted by data collectors for all applicable questions in order to minimize response bias during the interviews.

## Conclusions

The current study revealed higher LLIN utilization and ownership compared to similar studies conducted in the country and in sub-Saharan Africa. However, it revealed that a substantial proportion of households did not use LLIN or they had no net at all. Besides this, more than 10% of LLINs in use had holes that can allow mosquito entry. Therefore, it is imperative to improve public knowledge on the importance of LLIN in malaria prevention through intensified promotion and health education. Assistance on appropriate hanging of the nets, and inspecting that a net is potent and usable are also urgently important. Future research works should be complemented by qualitative study to investigate reasons of non-use and possible misuse of LLINs.
